# INO80 is Required for Osteogenic Differentiation of Human Mesenchymal Stem Cells

**DOI:** 10.1038/srep35924

**Published:** 2016-11-02

**Authors:** Chenchen Zhou, Jing Zou, Shujuan Zou, Xiaobing Li

**Affiliations:** 1 State Key Laboratory of Oral Diseases, West China Hospital of Stomatology, Sichuan University, Chengdu, China; 2 Department of Orthodonitcs, West China School of Stomatology, Sichuan University, Chengdu, China

## Abstract

Bone marrow derived human mesenchymal stem cells (MSC) are a great source in bone tissue engineering. However, how to improve the efficiency of MSC osteogenesis remains a big challenge in bone regenerative medicine. Here, we characterized the role of INO80 chromatin remodeling complex in osteogenic differentiation of MSC. We showed that silencing of subunits of INO80 reduced the mineral deposition of MSC in osteogenic condition. Moreover, INO80-silencing MSC cultured in osteogenic condition expressed lower mRNA levels of osteoblast-specific genes, including Runx2, Osx, Col1α1 and OCN. INO80 can interact with Wdr5 in MSC and positively regulates the canonical Wnt signaling transduction. Importantly, the mice implanted with INO80-silencing MSC displayed less bone formation. Overall, our study provides a new mechanism regarding osteogenic differentiation of MSC and could potentially be applied in clinical tissue engineering and treatment of osteoporosis.

Bone marrow derived mesenchymal stem cells (MSC) have been regarded as an excellent choice for cell-based tissue engineering therapy for bone^1^. Current strategies include the use of MSC, scaffolds, growth factors, or a combination of the three. However, how to improve osteogenic differentiation efficacy remains as one of the most challenging aspects for this therapy. Epigenetic mechanisms, such as DNA methylation, histone modification, expression of non-coding RNAs and chromatin remodeling, play a central role in the activation of proper transcriptional pathways during various biological processes, including MSC maintenance and lineage differentiation^2^,^3^,^4^. For example, promoters of early developmental genes in MSC often display DNA hypermethylated pattern, whereas lineage-specification genes are hypomethylated^5^. In addition, the status of histones H3 and H4 acetylation paired with the chromatin remodeling activities to induce the expression of the bone-specific osteocalcin (OC) gene^6^. Moreover, overexpressing of histone deacetylase 4 (HDAC4) in synovia-derived stem cells can promote and maintain chondrogenesis mediated by TGF-beta1^7^. Furthermore, long non-coding RNAs (lncRNA) are also essential in regulating MSC lineage differentiation. Recent study has demonstrated that HoxA-AS3 can be associated with EZH2 and direct the lineage specification of MSC^8^, implying multiple epigenetic mechanism are involved in regulation of MSC differentiation.

The INO80 chromatin-remodeling complex is critical in regulation of transcriptional activation and repression. In fact, the identification of INO80 gene is based on its ability to regulate inositol-responsive gene expression^9^. In mammals, INO80 complex can be associated with YY1 and involved in cell growth, cell-cycle control, proliferation, differentiation and apoptosis^10^. More importantly, INO80 complex plays an essential role in embryonic stem cells (ESC) self-renewal, somatic cell reprogramming, and blastocyst development^11^. INO80 complexes can function in several different types of nuclear transactions, including transcriptional regulation, DNA repair and DNA replication. Specifically, INO80 complex mediate the transcriptional activation of the pluripotency genes via interaction with core transcriptional regulatory circuitry^11^, indicating a role of INO80 complex in stem cell function. However, study regarding INO80 in MSC lineage specification has not yet been reported. WD repeat domain 5 (Wdr5), a key component of the mammalian Trithorax (trxG) complex, can function as an effector of H3K4 methylation and regulate stem cell activities^12^. However, the role of Wdr5 in MSC lineage specification and its relationship with INO80 in MSC are largely uncharacterized.

To evaluate the effect of INO80 on osteogenesis of MSC, we transfected MSC with siRNAs targeting INO80 and measured their osteogenic capability. We have also monitored the expression osteogenic markers, including Runx2, Osx, Col1α1 and OPN, of these MSC during their *in vitro* osteogenic induction. We identified Wdr5 acted as a partner of INO80 in MSC. Both INO80 and Wdr5 are responsible for canonical Wnt signaling transduction in MSC. Finally, we have analyzed bone formation of MSC *in vivo* when INO80 or Wdr5 were silenced. Our data uncovered an important role of INO80 in MSC osteogenic differentiation and provide new insights into the molecular mode of action of INO80 in regulating MSC lineage commitment.

## Materials and Methods

### Ethics

All experimental protocols and procedures were approved by State Key Laboratory of Oral Diseases, West China Hospital of Stomatology, Sichuan University. The animal procedures were conducted in accordance with *The Guidelines for the Care and Use* of Laboratory Animals of State Key Laboratory of Oral Diseases, West China Hospital of Stomatology, Sichuan University.

### Cell culture and osteogenic differentiation

Human bone marrow-derived mesenchymal stem cells (MSC) were purchased from ATCC (PCS-500–012) and cultured in Dulbecco’s modified Eagle’s medium (DMEM) containing 10% fetal bovine serum (Invitrogen, China), 100 IU/ml penicillin and 100 μg/ml streptomycin (Gibco, China), 2 mM l-glutamine (Gibco, China) at 37 °C in a humidified incubator with 5% CO_2_ in air. For osteogenesis, MSC were cultured with an osteogenic induction media containing 50 mg/ml ascorbic acid and 10 mM β-glycerophosphate sodium (Sigma-Aldrich, China)^13^. Media were changed every two days. siRNAs were added to the medium every 7 days during osteogenic induction.

### siRNA knockdown, lentivirus-mediated shRNA knockdown of MSC

All siRNAs targeting INO80 subunits and scrambled siRNA were obtained from Santa Cruz Biotechnology. Each siRNA consists of pools with three to five target-specific 19- to 25-nucleotide siRNAs designed to knock down target gene expression. For siRNA-mediated knockdown, MSC were transfected using LipofectamineRNAimax (Invitrogen, China) following the manufacturer’s instructions. One day after transfection, the medium was changed to culture medium or osteogenic medium. RNA was extracted 72 hrs after start of transfection. Knockdown was estimated by quantitative RT-PCR. For shRNA-mediated knockdown, the INO80 and Wdr5 shRNA expression lentivirus were purchased from Shanghai GeneChem Co., and the target shRNA sequences are the same as siRNA. Neomycin-resistant clones were selected with 0.5 mg/mL G418 (Invitrogen) for 10 days. Stably transfected clones were analyzed for expression of INO80 or Wdr5 via quantitative RT-PCR.

### Gene expression analysis

Total RNA was isolated from MSC at different time points using TRIZol Reagent according to the manufacturer’s instructions (Thermo Fisher Scientific, China). cDNA was synthetized from 2 μg of total RNA using QuantiTec reverse transcription kit (Qiagen, Valencia, CA, USA)^14^. Real-time polymerase chain reaction (PCR) was performed on a 7500 Real-Time PCR System (Applied Biosystems, China). Genes of interest were detected using specific oligonucleotide primers as specified in Supplementary Table 1. Glyceraldehyde-3-phosphate dehydrogenase (GAPDH) was used as an internal control.

### Alizarin Red S (ARS) staining

For ARS staining, MSC were fixed with 3.7% formaldehyde for 10 min followed by PBS rinse twice at room temperature. The cells were then stained with 1% Alizarin red S (pH 4.2, Sigma-Aldrich) for up to 5 minutes at room temperature^15^. Images of MSC stained with ARS were captured and stored digitally. Calcium deposition was measured by eluting ARS staining with 20% methanol, 10% acetic acid and 70% distilled water at room temperature for 10 minutes. The absorbance of the supernatants was measured at 405 nm. Relative ARS staining was then shown as a fold change of the control.

### Immunoprecipitation and Western blot analysis

MSC were isolated in radioimmunoprecipitation assay buffer (RIPA buffer). For IP, the lysates were immunoprecipitated using protein A beads mixed with 2 μg of normal rabbit IgG, INO80 or Wdr5 antibody at 4 °C overnight. The beads were washed and eluted. Input and IP samples were resolved on a SDS-PAGE gel, transferred onto PVDF membrane, and immunoblotted with specific antibodies as shown in the figure legends. Antibodies information is listed as below: INO80 (1:1000, ab22238, Abcam, China), Wdr5 (1:1,000, ab56919, Abcam, China). All of the IP experiments were repeated at least three times.

### Xenograft model for MSC-mediated bone formation

2 × 10^6^ MSC from different groups were mixed with 40 μg of tricalcium phosphate/hydroxyapatite (TCP/HA) powder (Sigma-Aldrich, China) and injected subcutaneously into immunodeficient mice (8–12 weeks old; Model Animal Research Center, Nanjing, China). Tissues were then harvested 28 days after injection and analyzed histologically.

### Statistical Analyses

All values are displayed as mean ± SD. Student’s paired t-test was used to identify significant differences between the scrambled and specific siRNA-transfected MSC at each time point as previously described^16^. A p < 0.05 was regarded as statistically significant.

## Results

### The INO80 complex is essential for MSC osteogenic differentiation

**Figure 1:**
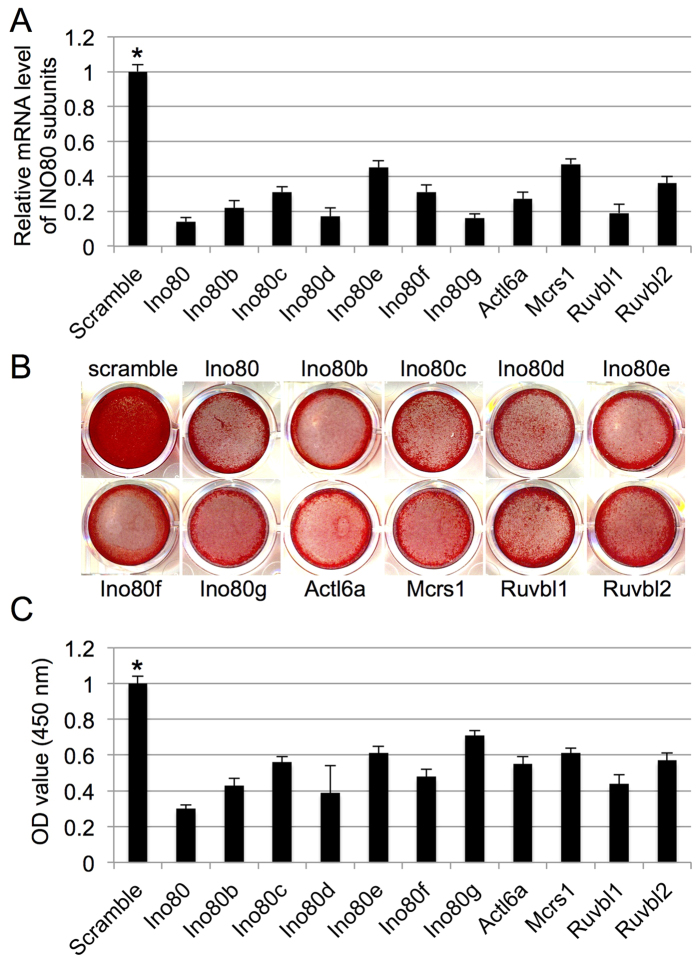
INO80 is required for MSC osteogenesis. (**A**) Quantitative RT-PCR results of the relative transcripts of INO80 subunits after siRNA transfection. Gene expression was normalized to GAPDH gene. (**B**) ARS staining of MSC treated with scramble siRNA or siRNA against different INO80 subunits and grown in osteogenic media for 28 days. (**C**) Quantification of Alizarin Red S stain via optic density (O.D.) measurement at O.D. 450 nm (n = 3). Results are shown as the relative fold changes to control MSC (mean ± SD), and significance is determined by Student’s t-test. (*p < 0.05).

**Figure 2:**
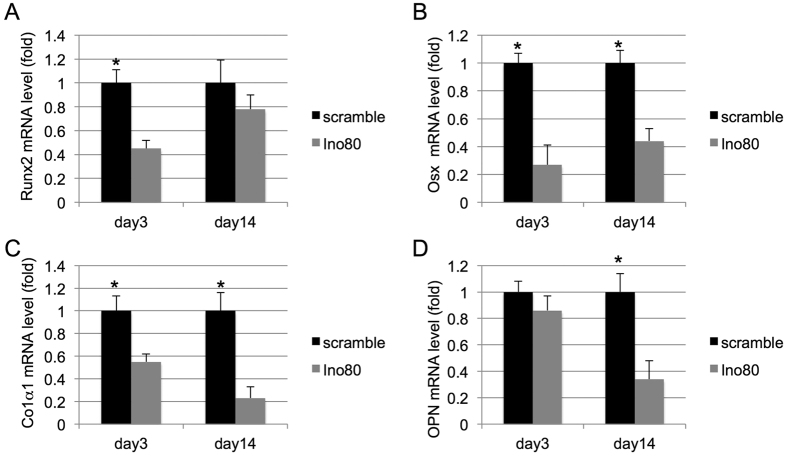
INO80 is required for osteogenic markers expression. (**A**) Runx2 is detected in control and INO80-silencing MSC in osteogenic condition from 3 days to 14 days. (**B**) Osx is detected in control and INO80-silencing MSC in osteogenic condition from 3 days to 14 days. (**C**) Col1a1 is detected in control and INO80-silencing MSC in osteogenic condition from 3 days to 14 days. (**D**) OPN is detected in control and INO80-silencing MSC in osteogenic condition from 3 days to 14 days. Results are shown as the relative expression to control MSC (mean ± SD), and significance is determined by Student’s t-test. (*p < 0.05).

To understand the role of INO80 complex in MSC osteogenic differentiation, we transfected MSC with a panel of siRNAs targeting each subunits. Results from quantitative real-time reverse transcription polymerase chain reaction (qRT-PCR) confirmed high efficiencies of gene silencing (Fig. 1A). Knockdown MSC were then cultured in osteogenic condition. To detect the deposited mineralized matrix in these MSC, we performed Alizarin Red S (ARS) staining after 21 days of induction. INO80 knockdown MSC displayed less intensive ARS staining than the control MSC (Fig. 1B). This was further validated by quantitative data acquired via measuring OD values (Fig. 1C). We detected no difference in expression level of INO80 during osteogenic induction of MSC (data not shown). We further examined the osteogenic differentiation using qRT-PCR. Decreased Runx2 levels were found on days 3 in INO80 knockdown MSC (Fig. 2A). Osx transcript levels were reduced on day 3 and 14 in INO80 knockdown MSC (Fig. 2B). Similarly, expressions of Col1α1 mRNA were dramatically inhibited on day 3 and 14 INO80 knockdown MSC (Fig. 2C). Furthermore, in INO80 knockdown MSC, we detected less transcript levels of OCN on day 14 (Fig. 2D). Thus, INO80 is required for MSC osteogenesis.

### INO80 interacts with Wdr5 in MSC

**Figure 3:**
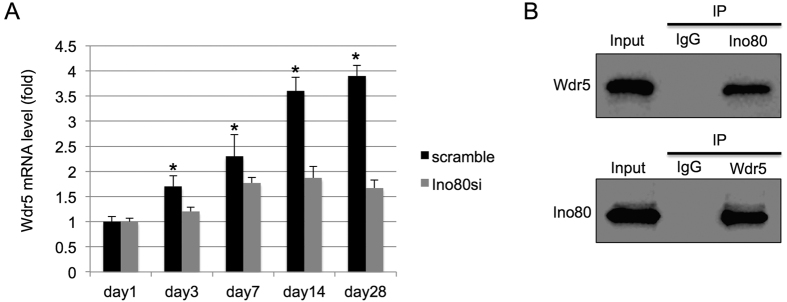
INO80 interacts with Wdr5 in MSC. (**A**) Wdr5 is decreased in INO80-silencing MSC compared with control MSC. (**B**) Interaction between INO80 and Wdr5. MSC lysates were sonicated and incubated with IgG or Ino80 or Wdr5 antibodies, and the presence of Ino80 or Wdr5 in the copurified proteins were detected with western blot.

In embryonic stem cells, INO80 is associated with Wdr5, which is important for skeletal development^11^,^17^. We reasoned that INO80 interacts with Wdr5 in MSC to regulate osteogenic differentiation. To test that possibility, we first examined the mRNA expression of Wdr5 during osteogenic differentiation, we found the Wdr5 steadily increased upon induction (Fig. 3A). However, this trend was abolished in INO80 silencing MSC (Fig. 3A). In addition, to explore whether INO80 interacts with Wdr5, we performed INO80 and Wdr5 immunoprecipitation assay. Wdr5 was detected in INO80 immunoprecipitates (Fig. 3B) and INO80 was also detected in Wdr5 immunoprecipitates (Fig. 3B), suggesting a role of Wdr5 in regulating osteogenic differentiation of MSC.

### Wdr5 is critical for MSC osteogenic differentiation

**Figure 4:**
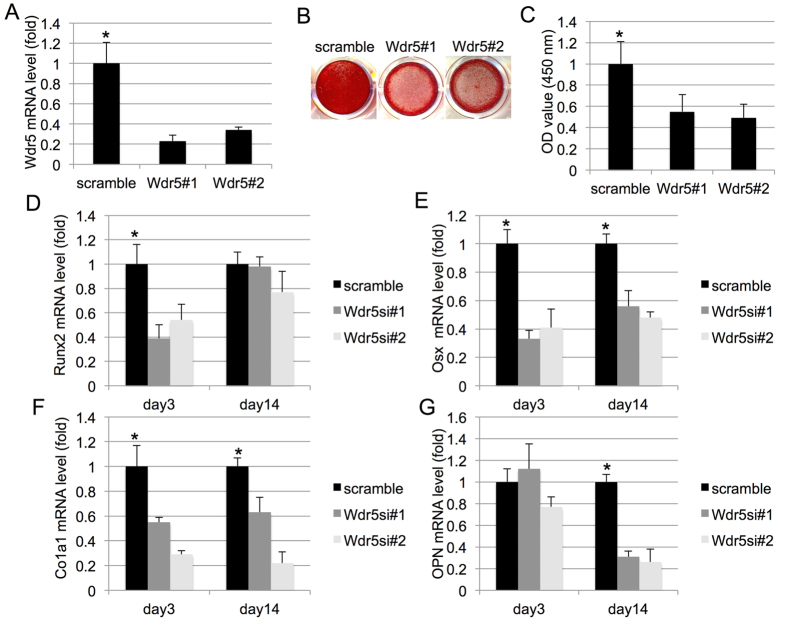
Wdr5 is required for MSC osteogenesis. (**A**) Quantitative RT-PCR results of the transcripts of Wdr5 after siRNA transfection. (**B**) ARS staining of MSC treated with scramble siRNA or siRNAs against Wdr5 and grown in osteogenic media for 28 days. (**C**) Quantification of Alizarin Red S stain via optic density (O.D.) measurement at O.D. 450 nm (n = 3). (**D**) Runx2 is detected in control and Wdr5-silencing MSC in osteogenic condition from 3 days to 14 days. (**E**) Osx is detected in control and Wdr5-silencing MSC in osteogenic condition from 3 days to 14 days. (**F**) Col1α1 is detected in control and Wdr5-silencing MSC in osteogenic condition from 3 days to 14 days. (**G**) OPN is detected in control and Wdr5-silencing MSC in osteogenic condition from 3 days to 14 days.

To investigate the role of Wdr5 in MSC osteogenic differentiation, we transfected MSC with two siRNAs targeting Wdr5. Knockdown of Wdr5 by siRNAs were confirmed by qRT-PCR (Fig. 4A). Knockdown MSC were then cultured in osteogenic condition and subjected for ARS staining after 21 days of induction. Wdr5 knockdown MSC displayed less intensive ARS staining than the control MSC (Fig. 4B), which was confirmed by quantitative data (Fig. 4C). In addition, we detected decreased of Runx2 levels on days 3 in Wdr5 knockdown MSC (Fig. 4D). Osx transcript levels were reduced on day 3 and 14 in Wdr5 knockdown MSC (Fig. 4E). Consistently, expressions of Col1α1 mRNA were inhibited on day 3 and 14 Wdr5-silencing MSC (Fig. 4F). Furthermore, in Wdr5-silencing MSC, we detected less transcript levels of OCN on day 14 (Fig. 4G).

### INO80 and Wdr5 are important for activation of canonical Wnt signaling

**Figure 5:**
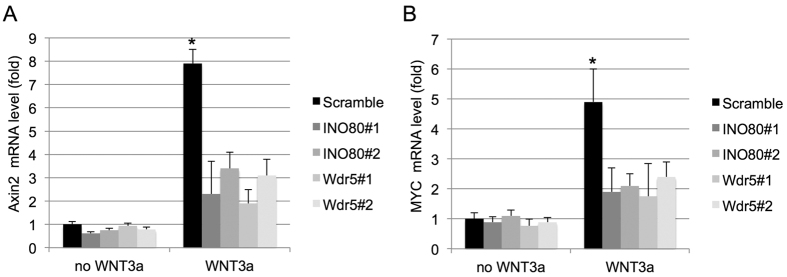
INO80 and Wdr5 are required for the expression of canonical Wnt target genes. (**A**) Quantitative RT-PCR of Axin2 was performed on mRNA isolated from MSC transfected with scramble siRNA or siRNAs against INO80 or Wdr5 cultured without or with Wnt3a. (**B**) Quantitative RT-PCR of MYC was performed on mRNA isolated from MSC transfected with scramble siRNA or siRNAs against INO80 or Wdr5 cultured without or with Wnt3a.

Previous studies have shown that Wdr5 is required for canonical Wnt signaling in osteoblasts^18^. To determine whether INO80 and Wdr5 were responsible for Wnt signaling activation in MSC, we treated MSC with Wnt3a for 4 hr and analyzed target genes activation of the canonical Wnt signaling pathway. The expressions of target genes, Axin2 and MYC, elevated dramatically upon Wnt3a treatment. However, the transcripts of Axin2 and MYC were significantly decreased at both INO80 and Wdr5 depleted MSC (Fig. 5A,B). Overall, these findings indicated both INO80 and Wdr5 are involved in canonical Wnt signaling.

### INO80 and Wdr5 are important for MSC-based bone formation *in vivo*

**Figure 6:**
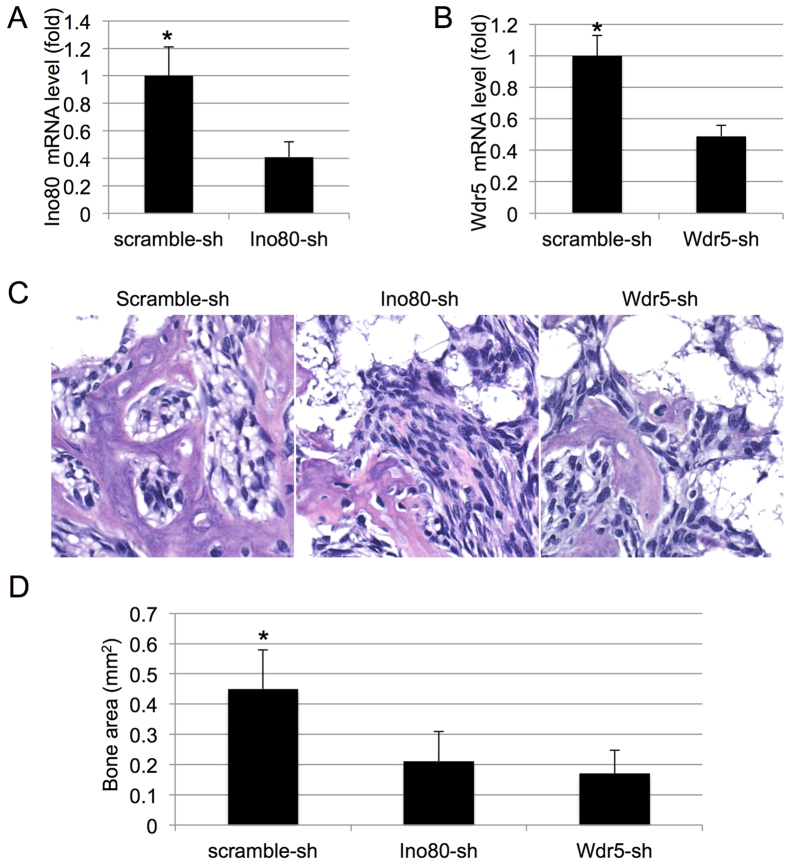
INO80 and Wdr5 are required for bone formation *in vivo*. (**A**) Quantitative RT-PCR results of the transcripts of INO80 after INO80 shRNA-virus infection. (**B**) Quantitative RT-PCR results of the transcripts of Wdr5 after Wdr5 shRNA-virus infection. (**C**) H&E staining of bone tissues from MSC with scramble-sh, Ino80-sh and Wdr5-sh. (**D**) Quantification of bone area from MSC with scramble-sh, Ino80-sh and Wdr5-sh.

To determine whether INO80 and Wdr5 play roles in MSC-mediated bone formation *in vivo*, we transfected MSC with lentivirus expressing shRNA against INO80 or Wdr5 and selected the stable lines with neomycin. Knockdown was validated by qRT-PCR (Fig. 6A). Control, INO80 or Wdr5 MSC were then mixed with TCP/HA and placed subcutaneously in the dorsal region of immunocomprimised mice. Bone tissues were dissected out from mice and analyzed after 28 days. We noticed less bone formation in INO80 or Wdr5 depleted groups than the control group by H&E staining (Fig. 6B). Measurement of the bone area showed that INO80 and Wdr5 knockdown led to decrease of bone tissue *in vivo* (Fig. 6C). Overall, the results of *in vivo* study showed INO80 and Wdr5 are important for MSC-based bone formation.

## Discussion

MSC in bone marrow have a multi-lineage potential and can give rise to osteoblasts, chondrocytes and adipocytes^19^. Decease in MSC number and osteogenic capability in the aging bone marrow causes age-related osteoporosis^20^. In current study, we evaluate the effects of INO80 on osteogenesis of MSC. We found that depletion of INO80 led to decease expression of RUNX2, OSX, Col1α1 and OPN. Furthermore, we found INO80 could interact with Wdr5 and mediate canonical Wnt activity. Our *in vivo* study recapitulated the findings from the *in vitro* experiments.

Epigenetic regulation of gene expression has an important role in normal and malignant tissue development, regeneration^4^,^21^,^22^.

The differentiation of multipotential MSC is under tightly epigenetic control at various levels, including DNA methylation, histone modification and chromatin remodeling^3^,^5^,^7^,^23^,^24^. In addition, component of chromatin remodeling SWI/SNF complex Brg1, is required for the expression of Alkaline Phosphatase (ALP), an early maker of osteoblast differentiation^25^. BRG1 can also be recruited to the promoter region OC and activate the expression of OC^26^. We identified a positive regulation of INO80 in MSC osteogenic differentiation. Components of SWI/SNF chromatin remodeling complex play important roles in osteogenesis of MSC. During osteogenesis, Osx can recruit Brg1 to the promoter of its target genes to form transcriptionally active complex^27^. Brg1 also participates in recruitment of C/EBPβ and RNA polymerase II to the promoter region OC^26^. Whether INO80 binds to the promoters of master transcription factors, such Runx2 and Osx, remain unknown.

Over the past decade, many studies have carried out to characterize the epigenetic control of MSC lineage differentiation. For example, involvement of histone acetylation in chondrogenic differentiation of MSCs has also been reported^7^. Intriguingly, a recent study showed that lncRNA HoxA-AS3 is required for adipogenesis of MSC^8^. HoxA-AS3 silencing in MSC lead to increase of osteogenic markers expression, including RUNX2, SP7, COL1A1, IBSP, BGLAP and SPP1^8^. HoxA-AS3 functions together with EZH2 to modulate the status of H3K27me3 in the promoter region of key osteogenic transcription factor Runx2^8^. This is an important study to provide evidence that MSC lineage commitment can be achieved through lncRNA regulation. Wdr5 is a core member of the mammalian Trithorax (trxG) complex. In ESC, Wdr5 can regulate the self-renewal via interaction with the pluripotency transcription factor Oct4^12^. Interestingly, INO80 is also associated with Wd5 and OCT4 and regulated ESC self-renewal and reprogramming^11^. In current study, we found that INO80 can be also associated with Wdr5 in MSC, suggesting a universal link between these two factors. Wdr5 is an important factor for osteoblast differentiation and skeletal development^17^,^18^. Moreover, Wdr5 can regulate both osteoblast and chondrocyte differentiation *in vivo*^28^. It seems likely that INO80 can occupy the same set of genes involving in osteoblast differentiation. However, whether this is the case required a systematic examination and Chip-sequencing can serve as a useful approach for this investigation. Previous report showed that in MC3T3-E1 cells, Wdr5 is required for activation of canonical Wnt target gene c-myc and inhibition of Wnt pathway repressor, sfrp2^18^. Similarly, we found Wdr5 is also required for Wnt activities in MSC. It is known that canonical Wnt signaling regulates many developmental processes and can promote osteogenesis^29^,^30^,^31^. We showed both INO80 and Wdr5 can positively regulate Wnt signaling transduction. However, whether INO80 can interact with key Wnt signaling transducer β-catenin remains to be identified.

In summary, our study showed that INO80 is required for promotion of MSC osteogenic differentiation and provides a valuable finding for bone tissue engineering. Moreover, INO80 can be presented as a new avenue to study MSC-related aging and osteoporosis *in vivo*.

## Additional Information

**How to cite this article**: Zhou, C. *et al*. INO80 is Required for Osteogenic Differentiation of Human Mesenchymal Stem Cells. *Sci. Rep.* **6**, 35924; doi: 10.1038/srep35924 (2016).

**Publisher’s note:** Springer Nature remains neutral with regard to jurisdictional claims in published maps and institutional affiliations.

## Supplementary Material

Supplementary Information
