# Supplementary material for: INO80 is Required for Osteogenic Differentiation of Human Mesenchymal Stem Cells
Source: Sci Rep. 2016 Nov 2;6:35924. doi: 10.1038/srep35924 (PMC5090198; doi:10.1038/srep35924)
Supplement: Supplementary Information [file srep35924-s1.pdf]

INO80 is Required for Osteogenic Differentiation of Human Mesenchymal Stem Cells

Chenchen Zhou [1],Jing Zou[1,2], Shujuan Zou[1,2], Xiaobing Li [1,2,*]

1 State Key Laboratory of Oral Diseases, West China Hospital of Stomatology, Sichuan University, Chengdu, China

2 Department of Orthodonitcs, West China School of Stomatology, Sichuan University, Chengdu, China

*: Corresponding author

Xiaobing Li

State Key Laboratory of Oral Diseases, West China Hospital of Stomatology, Sichuan University

14 Third Section, Renmin Nan Road, Chengdu 610041, China.

E-mail address: lxb_30@hotmail.com

Tel: +86 18628031391

Supplementary Table 1 Primers and shRNA sequences

Realtime RT-PCR primers

INO80 F	5'-TGCGACAAACGTCAGCTATCT-3'
INO80 R	5'-CTGGGGCAATAATGGATTACTGT-3'
INO80b F	5'-AGCCTCAGCTCAAACTCAAAAT-3'
INO80b R	5'-TTATCCACAACCATAAGGGGAGA-3'
INO80c F	5'-TCGCTTCTGAAAGGGCATTG-3'
INO80c R	5'-CTTAGCTGGCTTAAAGGATGGAG-3'
INO80d F	5'-ATAAGCCCTTGTGCTCATATAGC-3'
INO80d R	5'-AGCGTTGGCTGTTATACTTGG-3'
INO80e F	5'-TACCGGAATCTGAAGCGGAAG-3'
INO80e R	5'-TCTAGGAGGAAACTCTTGTCCC-3'
INO80f F	5'-GCGTTGCCTCCCCTATTTG-3'
INO80f R	5'-CTGCGATTTAATTCCCGCTGG-3'
INO80g F	5'-GAGGCACCAGAGTTAGTCTGC-3'
INO80g R	5'-GGAGGTGTTCACGTTGAGAATC-3'
Actl6a F	5'-TGGAGGCCATTTCACCTCTAA-3'
Actl6a R	5'-TCTTTGCTCTAGTATTCCACGGT-3'
Mcrs1 F	5'-GGGCTGCTAGATTCATCCCTG-3'
Mcrs1 R	5'-GGAGGAGCTTCTCCGTTTAGG-3'
Ruvbl1 F	5'-AGGTGAAGAGCACTACGAAGA-3'
Ruvbl1 R	5'-CTACTATGACGCCACATGCCT-3'
Ruvbl2 F	5'-GTCGGGCAGTCCTTATTGCT-3'
Ruvbl2 R	5'-TGGTCGATCAATCTGGATCTCC-3'
Runx2 F	5'-TGGTTACTGTCATGGCGGGTA-3'
Runx2 R	5'-TCTCAGATCGTTGAACCTTGCTA-3'
Osx F	5'-CCTCTGCGGGACTCAACAAC-3'
Osx R	5'-AGCCCATTAGTGCTTGTAAAGG-3'
Col1a1 F	5'-GAGGGCCAAGACGAAGACATC-3'
Col1a1 R	5'-CAGATCACGTCATCGCACAAC-3'
Opn F	5'-CTCCATTGACTCGAACGACTC-3'
Opn R	5'-CAGGTCTGCGAAACTTCTTAGAT-3'
Wdr5 F	5'-AATTCAGCCCGAATGGAGAGT-3'
Wdr5 R	5'-AGGCTACATCGGATATTCCCAG-3'
Axin2 F	5'-CAACACCAGGCGGAACGAA-3'
Axin2 R	5'-GCCCAATAAGGAGTGTAAGGACT-3'

Myc F	5'-GGCTCCTGGCAAAAGGTCA-3'
Myc R	5'-CTGCGTAGTTGTGCTGATGT-3'
shRNA sequence	
Ino80	5'-GGGAAAUCCUCAUGAUAGAUU-3'
Wdr5	5'-CGAAAGAGAUUGUACAGAA-3'
